# The Ethical Complexity of Medical Decision Making in the Adolescent Oncology Patient

**DOI:** 10.3390/curroncol31080310

**Published:** 2024-07-24

**Authors:** Ariel Paige Nash, Avis Harden, Rachna Kalapi Sheth

**Affiliations:** MD Anderson Children’s Cancer Hospital, Houston, TX 77030, USA; anash@mdanderson.org (A.P.N.); aharden@mdanderson.org (A.H.)

**Keywords:** aMDM—adolescent medical decision making, SDM—shared decision making, pediatric oncology, adolescent and young adult (AYA)

## Abstract

Adolescent Medical Decision Making (aMDM) is frequently discussed but presents a significant challenge in practice, especially in cases of adolescents with life threatening or life limiting illnesses. In this paper, we present a case that explores the importance of aMDM, the difficulties for providers when engaging adolescents in these discussions, and how certain skills may be incorporated into pediatric practice. Literature suggests that patients of this age group, while being legally without capacity, have meaningful insights into their care. However, unless physicians feel comfortable and competent engaging adolescents in a manner that honors their developmentally appropriate understanding of their illness, these insights can be lost.

## 1. Introduction

Imagine an encounter with a developmentally normal 6-year-old patient with metastatic neuroblastoma presenting with opioid-induced constipation. The next steps are quite standard; start a bowel regimen. If the child refuses, discuss ways to encourage adherence with the parents using a shared decision-making (SDM) mindset. This may include escalation of care to inserting a nasogastric tube. Now, imagine the patient is 16 years old. How might your approach change? And how should it—considering the patient’s age and evolving capacity to make independent decisions?

Throughout the United States, children under 18 years old require parental consent for most medical treatments or procedures [1,2]. These legal guidelines do not always result in the most ethically satisfying outcomes, especially regarding adolescents and patient autonomy. Meaningful assent and parental authority can compete and lead to distressing challenges when it comes to the care of adolescent patients, especially when they are on the verge of legal adulthood [1,3]. To complicate matters, these patients may have serious life-threatening illnesses and/or be financially and psychosocially dependent on their caregiver. One tactic to address this problem is to allow adolescents to directly engage in their medical decision making by encouraging active participation rather than passive observation [4].

In this discussion, we present a case demonstrating the complicated nature of adolescent medical decision making (aMDM) in a chronic, life limiting illness. We define aMDM as direct involvement of adolescent patients in decisions involving their medical care. We hope to illustrate the difficulties in engaging adolescents, how this contributes to provider distress, and how direct and deliberate use of aMDM by providers improves care. The patient’s name and some identifying information have been altered to protect the patient’s identity.

## 2. Case of an Adolescent Newly Diagnosed with Carcinoma

“Lucy,” an 18-year-old young woman, was admitted with a solid tumor carcinoma that had metastasized to several sites including organs and bone. She was diagnosed six months prior at 17 years old after presenting with several months of back pain. Her mother had been the primary decision maker throughout her care, with minimal input from Lucy in medical discussions. Within six months, she had progressed through multiple lines of therapy and was being considered for additional rounds of traditional chemotherapy or an experimental treatment. She remained admitted for poorly controlled pain requiring increasing amounts of medication.

Her mother continued to be the primary decision maker, despite Lucy recently turning of legal age. Support services including palliative care and psychology attempted to meet with Lucy but she often deferred. As potential next steps and therapies were discussed, her mother strongly expressed that conventional therapy had failed her daughter and going forward she was interested exclusively in homeopathic methods of treatment. During these meetings Lucy remained reserved and did not participate in the conversation, deferring all decisions to her mother.

Before any treatment decisions could be made and after prolonged tensions between Lucy and her mother, the two were heard having a heated argument ending with her mother leaving. On further conversation with Lucy, she reported that they argued about the homeopathic methods because Lucy herself was more interested in an experimental treatment offered by her oncologist. Although Lucy had been informed of her legal rights as an adult, she battled with significant guilt about the disagreement and felt uncertain she could make an independent decision.

## 3. Adolescent Medical Decision Making (aMDM)

At this point in her care, Lucy had arrived at a crossroads; she was transitioning from adolescence to young adulthood and being asked to make medical decisions with significant long-term impact. This time of adolescence, defined by the WHO as individuals aged 10–19 years old, can be tumultuous as the neurobiological, psychological, and even legal development of these patients is highly variable [5,6,7]. For the purposes of this paper, we will define adolescence from ages 10–21 years-old [3]. Medical decisions historically are granted to the parent when the adolescent is less than the age of 18, assuming parents will act in their best interest [1,8]. The AAP has written about this “best-interest” standard and has recommended a more realistic framework through the harm principle. In this, the goal is to identify a threshold at which parental decisions become harmful and outside intervention is indicated to protect the child [2]. It is recommended that adolescents have some input through assent appropriate to their developmental stage [1,2]. Despite these recommendations, efforts in shared decision making have largely focused on the input of the parent(s) and not the child [9]. Even in end-of-life conversations, the adolescent patient is often overlooked [10]. This oversight ignores the need for adolescent engagement in managing life threatening or life limiting diseases. Adolescents are rarely allowed to make medical decisions without parental input, except in specific situations such as pregnancy, treatment of sexually transmitted infections, or substance use disorders [11]. However, ethically, the meaningful assent of an adolescent with evolving capacity should be obtained. Unfortunately, even with efforts to share in medical decision making, the literature suggests that this has been largely unsuccessful [1,10].

Part of the challenge with aMDM is that an adolescent’s capacity for responsible decision making is poorly defined. Adults are legally implied to have capacity, by which they work with their physician to understand the risks and benefits of different treatments. Adolescents under the age of 18 in the United States, on the other hand, are assumed to lack capacity by legal standards. Interestingly, these same adolescents may be held criminally responsible under different legal standards. One study evaluated the differences in the minimum age for mental health consent versus the minimum age of criminal responsibility. Almost 90% of all countries evaluated allowed criminal prosecution for children and adolescents [12]. These same countries typically had laws that did not allow for medical consent earlier than 16–18 years of age.

The process of responsible decision making requires the same level of developmental maturity for decisions with legal consequences as with medical consequences. Decision making requires choosing between two or more alternatives, while considering the short- and long-term effects [13]. Certain core skills must be mastered, such as creative problem solving, compromise, and commitment [7]. Adolescents and young adults develop this risk analysis ability on a sliding scale, but this does not inherently make them incapable of making appropriate decisions [7]. The AAP considers capacity to be task-specific rather than an all-or-nothing phenomenon [2]. One prominent study almost 40 years ago showed that 14-year-olds did not differ significantly from 18- or 21-year-olds in expressing reasonable preferences regarding medical treatment in a controlled, hypothetical setting [14]. Other studies show that adolescents are capable of making decisions as an adult would, but when faced with time pressure or stress, they are more easily affected by peers, less future-oriented, and more impulsive than adult counterparts [11,15]. Some countries allow adolescents under 16 to consent to medical care by passing a competency test. In the UK and Scotland, if this test is passed, parents are not allowed to overrule the competent adolescent’s consent [7,12]. Acknowledging the potential of an adolescent to responsibly make difficult medical decisions is important to respecting their autonomy, especially as literature shows that adolescents want to be involved in difficult medical decisions, even when emotionally taxing [7].

Lucy and her mother had several choices to consider—conventional chemotherapy vs. experimental treatment, and supportive measures including hospice. Her mother brought in a new variable of an exclusively homeopathic treatment, not supported by Western science, and not necessarily preferred by Lucy. Due to her physical, emotional, and psychosocial dependence on her mother, Lucy felt conflicted despite reaching legal age and being granted the legal ability to make independent decisions. Without her mother’s support or previous experience making even minor decisions during the medical journey, Lucy felt unpracticed and reasonably worried that any decision she made could have large, unforeseen emotional and financial costs. This unmitigated conflict was a huge barrier to care and honoring her choices.

## 4. The Provider’s Role in aMDM

As Lucy’s hospitalization continued, her care team worked on guiding her to comfortably make decisions for herself. While she still felt paralyzed making choices about her cancer treatment, she started to engage in smaller decisions. She was encouraged to voice her opinions, such as trying an enema or another round of oral medications during a bout of significant constipation. Lucy was provided the space to make her own decisions without judgement and her choices were respected. Her providers included Lucy in conversations about her care and assured her that her wishes would be honored. This built not only her confidence but also a new support system during her hospitalization. An integral part of her developing these new skills was her care team’s effort in presenting these opportunities. Witnessing Lucy’s increased engagement and improved symptom management also provided satisfaction and peace to those who were caring for her.

Physicians, particularly in pediatrics, are expected to discuss medical decisions with parents and patients of varying developmental abilities while helping families navigate the medical system through a stressful time. This journey can strain the parent–child relationship and the therapeutic relationship [3]. Providers also have a responsibility to elicit an adolescent’s participation in health-related decisions, rather than the patient simply spectating [3,4]. The AAP toolkit for wellness visits recommends a questionnaire which directly engages adolescents about health and home concerns without their parents present to ensure privacy and safety [16]. This honors the ethical principles of autonomy and assent.

Lucy’s transition to decision maker was hardly a seamless, stepwise process. In practice, it was much more complex and difficult. During the months from diagnosis and leading up to Lucy’s 18th birthday, she made few medical decisions, as her mother was the primary decision maker. During family meetings, providers observed their relationship to be largely antagonistic. Lucy had not been confident in expressing her opinion during discussions with her mother present. The few times Lucy’s preferences were elucidated, they were often negatively received by her mother. After the abrupt departure of her mother at the time of her legal transition to adulthood, Lucy’s minimal participation caused concern, despite no neurological basis to question her capacity.

Lucy also had varying degrees of trust amongst the multiple subspecialties involved in her care, creating discordance in how each team assessed her ability to have meaningful discussions about her medical situation. Providers struggled with whether they should continue to work with Lucy individually or help reconcile her relationship with her mother. Clinical psychology assessed Lucy and had no concerns about her developmental ability to make decisions. The primary team was eventually able to establish enough trust with Lucy to have clear, defined conversations with her and her sibling as her chosen confidante and advocate. Despite this progress, when her mother suddenly reentered the conversation and hospital, Lucy reverted to her prior disposition, deferring all decisions back to her mother. The primary team attempted shared decision making between Lucy and her mother through multiple family meetings, but Lucy remained tight-lipped. Ultimately, the outcome of these meetings was the decision to return home with hospice.

The team had presented multiple options to Lucy as valid, and deeply supported her decisions. However, there was concern that Lucy’s autonomy had been overridden by her mother, especially as her mother initially refused to involve the supportive care team. The goal was for Lucy’s decisions to be in line with her own values, rather than a reflection of her mother’s or providers’ opinions. Within the harm principle’s framework of choosing an acceptable, medically reasonable treatment without actively causing harm, the team tried to advocate for Lucy. In one of the last moments with her providers, Lucy stated “I would have been for [further medical treatment], but you know how my mom is.” She faced an incredibly difficult decision at a time when she was vulnerable and dependent, but legally an adult. Ultimately, Lucy made the decision that would give her comfort, which included being surrounded by family in her last moments, along with her mother’s love and support.

## 5. Breaking through Barriers in aMDM

Research has suggested that shared decision-making interventions have not succeeded in bringing adolescents into medical conversations, especially at the end of life [1,9,10]. One barrier is providers’ lack of confidence or tools to feel comfortable engaging adolescents or emerging adults on their own [10,15]. There is also the desire to shield adolescent patients from distress, both from parents and the providers [10]. Adolescent medical decision making requires open conversation with a person whose thoughts may differ from the parents or even the providers, and the emotional fallout can be devastating. Moral distress is prevalent in providers as they engage in such conversations and can lead to compassion fatigue, burnout, and resignation [17]. One study surveyed pediatric oncology health care professionals about sources of moral distress and elicited common themes such as non-disclosure of prognosis to the child, conflicting goals of care, and witnessing a violation of standard procedures or ethics [17]. While these feelings of hopelessness can discourage providers from engaging adolescents, this study concluded that improved communication can alleviate this moral distress [17]. Other sources have also emphasized the importance of family meetings to allow all parties to express goals and repair breakdowns in communication [1,18].

We would argue that deliberately engaging adolescents, eliciting their opinion, and encouraging their own decision making can also relieve some of the moral distress of providers. There are resources available for providers to develop their skills for improved shared decision making and engagement with adolescents [18,19]. Drs. Sawyer and Rosenberg provide a helpful stepwise approach to these conversations, which we used to summarize this case in Table 1. It emphasizes working with the adolescent to practice small decisions which may build confidence for more difficult ones. Psychology or supportive care’s involvement is recommended in high-emotion situations [18]. Clinical child psychologists can assess an adolescent’s psychological and developmental abilities. The WHO has a tool for assessing an adolescent’s capacity that can be integrated into care [5].

When it comes to end-of-life conversations, having a well-established rapport with the patient is invaluable and arguably necessary [10]. This can come regardless of the discipline of the provider and is dependent on the effort to develop a connection. There should be emphasis on palliative care education for providers, such as Vital Talk, though this is mainly adult patient-oriented [10,20]. “Voicing my CHOiCES” is an evidence-based tool that can guide end-of-life conversations in adolescents [21].

Other sources also recommend asking the adolescent how they want to engage in such conversations [1]. Unfortunately, few validated tools or frameworks exist to guide adolescent and young adult (AYA) patients through the murky waters of evolving autonomy, capacity, and consent when facing a life threatening or life limiting illness. A multidisciplinary team at Dana-Farber developed a tool called MyPref, which they tested in 15 AYA oncology patients, aged 15–30, and found promising results [22]. Further such initiatives and research are sorely needed in this population and may serve as the basis to direct legal protections for the competent minor.

Adolescents may be notoriously difficult to engage, but as our experience with Lucy demonstrated, developing a relationship over time can improve communication and increase trust. Providers and parents should allow room for evolving capacity as the adolescent becomes more informed and experienced [1,5]. Whereas, early in adolescence, a parent is more likely to give direction or directives, this can evolve into more guided advice or an exchange about treatment options between the patient and parent [5]. There should also be the opportunity for the adolescent to have confidential conversations with their physician [10]. Ultimately, the parent has final, legal authority if the patient is under age 18, but coercing an adolescent into treatment options that go against their values or priorities can be damaging to the family unit through loss of trust and psychological harm.

## 6. Conclusions

Scenarios such as Lucy’s are inevitable for those of us who care for adolescents and young adults. We hope to call attention to these situations as opportunities for providers to exercise their role in aMDM. By initiating this practice early with “low risk” decisions, we believe adolescents can develop the confidence and skills to rely on when more difficult challenges arise down the road. Even if they ultimately defer decisions to their parents, we believe it will assuage some of the moral distress of providers.

In Lucy’s case, we felt we missed an opportunity of earlier engagement. If she felt her choices were heard and her strengths acknowledged, whether they had aligned with her mother’s opinion or not, she may have had increased confidence in decision making and clearer communication when her mother left. Engaging adolescents in decision making not only has potentially positive effects on provider distress and medical decision making, but it honors the autonomy of our adolescent patients at a time when they are finding their voice.

## Figures and Tables

**Table 1:** A stepwise approach to adolescent shared decision making (SDM). * As adapted from Sawyer and Rosenberg, AMA Journal of Ethics, May 2020.

Point in Shared Decision Making (SDM) *	Lucy’s Case	Recommendations *	Ideal Scenario
**Step 0: Prior to decisions**	The hospitalist and oncology team developed a strong relationship with Lucy	**Get to know the patient**	Team assembles early to establish relationship with Lucy and her mother at diagnosis
	First opportunity to make medical decisions while hospitalized and at end of life	**Set expectations for SDM**	Discuss shared decision-making goals and establish plan for when Lucy turns 18 years old
	Offered Lucy autonomy over decisions such as bowel and pain regimens	**Engage the patient in smaller choices**	Early expectations set by obtaining Lucy’s assent and input in all medical decisions
	Supportive care and psychology consulted early	**Consider consulting palliative care and other support services, such as clinical psychology**	Consult services early and build rapport
**Step 1: When making decisions**	Medically reasonable options were discussed such as continued treatment and hospice	**Define medically reasonable options with the adolescent and parents/guardian**	Allow solo discussions with Lucy regarding medically reasonable options in addition to family meetings
	Lucy and mother had conflicting goals of care and Lucy’s opinions were unclear to the team	**Honor medically reasonable decisions and give recommendations, if appropriate**	Lucy engages with providers in making medical decisions and has clearly defined goals of care
	Recognized intense emotions, especially frustration with current medical options	**Acknowledge emotions**	Acknowledge Lucy’s emotions first then create space for family discussion and input
	Lucy was inconsistently participatory	**Allow different levels of SDM with different families**	Lucy participates more readily and frequently in family meetings
**Step 2: When SDM becomes difficult**	Multiple attempts to engage in SDM with family to bring about unified decision were unsuccessful	**Always strive to maintain a therapeutic relationship and be flexible while maintaining patient safety**	Disagreements acknowledged and validated for both Lucy and her mother/family
	Hospice pursued but unclear if all her values were honored	**If the patient and parent(s) disagree about the best treatment plan and both preferences are medically reasonable, reengage in SDM**	Any conflicts are mitigated through SDM with providers
	Lucy had legal authority but felt unable to make decisions because of her dependence on her mother	**Acknowledge who has legal authority for a final decision, parent(s)/guardian in the case of a minor**	Lucy’s decisions, including end-of-life choices, are made in line with her own values, without burdensome guilt or undue pressure from providers and/or family
